# Prognostic and immunomodulatory roles of schizophrenia-associated genes HTR2A, COMT, and PRODH in pan-cancer analysis and glioma survival prediction model

**DOI:** 10.3389/fimmu.2023.1201252

**Published:** 2023-07-26

**Authors:** Jing Shen, Qiang Wang, Fengquan Lu, Hua Xu, Peng Wang, Yu Feng

**Affiliations:** ^1^Medical laboratory, The Affiliated Jiangsu Shengze Hospital of Nanjing Medical University, Suzhou, China; ^2^Suzhou Key Laboratory of Neuro-Oncology and Nano-Bionics, Suzhou, China; ^3^Medicine and Health, The University of New South Wales, Kensington, NSW, Australia; ^4^Melbourne Medical School, The University of Melbourne, Parkville, VIC, Australia

**Keywords:** schizophrenia, HTR2A, COMT, ProDH, glioblastoma and low-grade glioma, pan-cancer, single-cell RNA sequencing

## Abstract

**Background:**

The shortened life expectancy in schizophrenia (SCZ) patients may be correlated with most cancers, yet there is heterogeneity in the studies examining these correlations. This study explored the expression of SCZ-related genes (HTR2A, COMT, and PRODH) in pan-cancer analysis. It helped to enhance the mechanistic understanding of the SCZ-cancer relationship and their immune mechanisms at the genetic level. Additionally, this study established a survival prediction model for glioblastoma and low-grade glioma (GBMLGG).

**Methods and results:**

SCZ-associated genes (HTR2A, COMT, and PRODH) were subjected to pan-cancer analysis. COX regression analysis and survival analysis were carried out for differentially expressed genes in multiple cancers, and finally, GBMLGG was derived as the focus for further detailed analysis. The immune scores and immune cell infiltration analyses were performed. All three genes were considerably linked with immune infiltration in GBMLGG, consistent with survival analysis. Based on the immunocyte analysis, it was observed that CD8^+^ T cells might be critically involved in the survival of GBMLGG. Genomic heterogeneity studies identified correlations of three genes with GBMLGG in tumor mutational burden (TMB) and mutant-allele tumor heterogeneity (MATH). HTR2A and COMT were significantly negatively correlated in TMB. Furthermore, it was found that HTR2A had a significant positive correlation with MATH, whereas PRODH had a significant negative correlation with MATH. Accordingly, a survival prediction model was constructed for GBMLGG using these three genes and clinical data, with better results obtained when evaluated in two separate datasets. Finally, gene expression validation and further immunocyte analysis were carried out in the single-cell RNA sequencing (scRNA-seq) data of glioma.

**Conclusion:**

SCZ-associated genes (HTR2A, COMT, and PRODH) were significantly differentially expressed in the carcinogenesis and survival of multiple cancers. The up or downregulation of gene expression varied across cancer types. In the GBMLGG analysis, upregulation of HTR2A and COMT was significantly positively correlated with carcinogenesis, while the opposite was noted for PRODH. Furthermore, a negative correlation was found between the upregulation of HTR2A and COMT and the survival of GBMLGG, and the opposite was also noted for PRODH. As reflected in the immunocyte analysis, abnormal expression of the three genes might be linked with CD8^+^ T cell infiltration, which might be critically involved in the survival of GBMLGG patients. The expression of HTR2A and COMT may inversely affect the efficacy of immunotherapy through the TMB pathway and further affect the prognosis of patient survival. The expression of HTR2A might positively indicate the degree of tumor heterogeneity through MATH and further affect the survival and prognosis of patients. The negative correlation of PRODH led to the opposite effect. Finally, the constructed survival prediction model demonstrated good predictive value, which was well validated in scRNA-seq analysis.

## 1 Introduction

Schizophrenia (SCZ) is a common and severe mental disorder with a global prevalence of approximately 1% (1). In the general population, the life expectancy of individuals with SCZ is 10 years shorter than those without SCZ (2). There is a wide range of reasons, including medical conditions, living status, economic conditions, side effects of antipsychotic drugs, and suicide (3, 4). Other studies have suggested that this is mainly related to a range of aging, metabolism, and inflammation-related diseases, such as cancer, diabetes, and others (5, 6).Of interest, the HTR2A gene (encoding 5-hydroxytryptamine receptor 2A [5-HT2A, also known as HTR2A]) is one of the serotonin receptors that are neurotransmitters with multiple actions, and there are numerous studies demonstrating the specific and significant correlation of this gene to SCZ (7–9). Serotonin, a known neurotransmitter, has recently emerged as a tumor growth factor for several human cancers through interaction with its receptors (5-HTR 1–7), which are widely expressed across a range of tissues (10, 11).

The COMT gene (encoding catechol-O-methyltransferase [COMT]), one of the major regulators of dopamine function in the prefrontal cortex, has become a pivotal determinant of SCZ-related cognitive dysfunction and response to antipsychotic drugs (12, 13). The Val->Met polymorphism (rs4680), common in the COMT gene, is linked with enhanced dopamine catabolism in the prefrontal cortex, which impairs prefrontal cognition and may increase the risk of SCZ (14). The several-fold decline of enzyme activity caused by the genetic variant Val158Met in COMT leads to the accumulation of potentially carcinogenic endogenous catecholestrogens and their reactive intermediates, increasing the risk of tumorigenesis (15). The 22q11DS deficiency has been recognized as a genetic subtype of SCZ. Therefore, genes in this region might be involved in susceptibility to the disease. A frequent deficiency observed in 22q11DS is the PRODH gene, which encodes proline dehydrogenase (PRODH). This enzyme is responsible for catalyzing the initial step of proline catabolism (16). Cancer cells often modify their metabolism to support uncontrolled proliferation, and proline is the only secondary amino acid in the body that is protein-rich. The proline degradation occurs in the mitochondria, involving an oxidative step catalyzed by PRODH/proline oxidase (PRODH/POX) (17). A significant association of this gene with breast cancer has been documented in an existing study (18), but studies examine its correlation with other cancers are lacking. The HTR2A gene-encoding serotonin receptor 5-HT_2A_ is an important regulator of cognitive function in the developing brain and plays a key neurotransmitter role in many physiological processes. The COMT gene-encoding COMT has been extensively studied with regard to many psychiatric phenotypes, and it influences the genetic regulation of dopamines in the prefrontal cortex. The dopamine system has been recognized to underlie the prefrontal cortex-dependent cognitive functions, such as attention and executive function. The COMT genotype may be associated with mood episodes and some brain cell activities. In addition, the PRODH gene-encoding PRODH, a mitochondrial enzyme degraded by proline, regulates cancer cell survival and apoptosis. Exploration of whether these genes (HTR2A, COMT, PRODH) may influence immune cell differentiation through the immune pathways would provide interesting and plausible evidence for the correlation between SCZ and cancers. To assess whether the TIME was affected by this pathway, the link between the expression of HTR2A, COMT, PRODH and the degree of immune cell infiltration in each cancer type was examined.

Significant research heterogeneity on the association between SCZ and most cancers has been noted, along with whether SCZ reduces life expectancy among patients with mental illness. For example, studies by Giovanni Perini et al. have supported a negative association between SCZ and cancer mortality (19–21). In contrast, Zhou et al. have highlighted the exact opposite conclusions by meta-analysis (22–25). Therefore, this study aims to delineate the association between SCZ-related genes (HTR2A, COMT, PRODH) and different cancers.

## 2 Materials and methods

### 2.1 Materials

The top three SCZ-related genes HTR2A (score = 43.45), COMT (score = 49.95), and PRODH (score = 38.36), ranked by Relevance Scores, were obtained from the GeneCards database (https://www.genecards.org/).

The standardized pan-cancer datasets: The Cancer Genome Atlas (TCGA), Therapeutically Applicable Research To Generate Effective Treatments (TARGET), Genotype-Tissue Expression (GTEx) (PANCAN, N = 19131, G = 60499) were downloaded from the University of California, Santa Cruz (UCSC) database (https://xenabrowser.net/). The expression data of HTR2A, COMT, and PRODH genes in each sample were extracted from these datasets. After performing a log-transformation on each expression value, cancer types with fewer than three samples were excluded from the analysis.

Finally, the study retrieved the expression data for a total of 34 types of cancer. The corresponding clinical information, such as patient age, gender, and tumor grade, was collected from the portal. The tumor mutational burden (TMB) and mutant-allele tumor heterogeneity (MATH) were also downloaded from the TCGA database.

**Figure 1:**
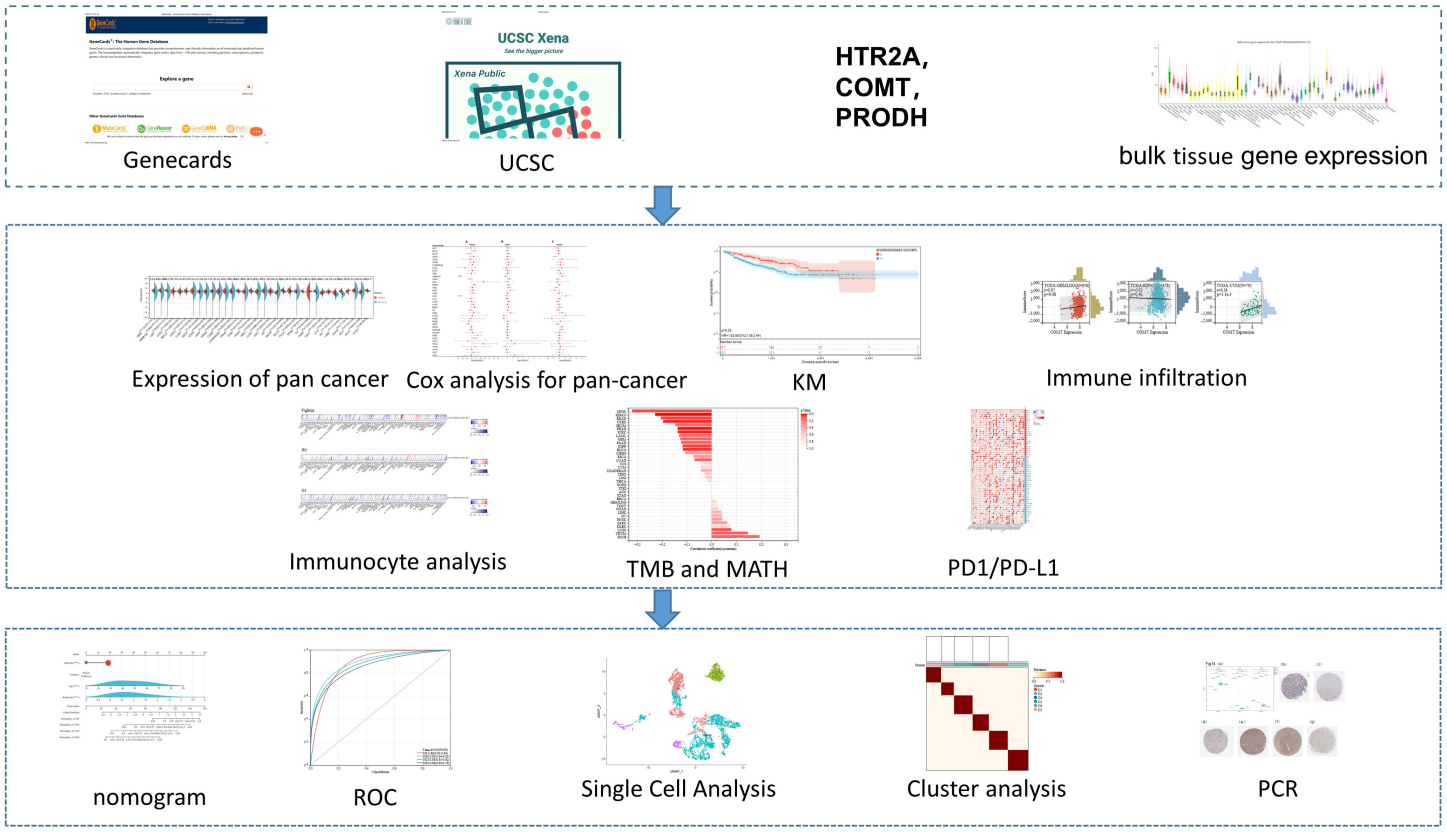
Flow Chart.

The study obtained the validation dataset CGGA.mRNAseq_693 and the single-cell RNA sequencing (scRNA-seq) dataset CGGA.scRNA_6148 from the Chinese Glioma Genome Atlas (CGGA) database (http://www.cgga.org.cn/). The data were then normalized. The flow chart is displayed in **Figure 1**.

### 2.2 Differential expression of HTR2A, COMT, and PRODH in various tissues

The expression levels of HTR2A, COMT, and PRODH in different human tissues were analyzed using data from different tissues of healthy individuals from the GTEx database.

### 2.3 Differential expression of HTR2A, COMT, and PRODH in various cancers

Utilizing the R software (version 3.6.4), the study measured the differences in expression levels of HTR2A, COMT, and PRODH between normal and tumor samples in each cancer based on data obtained from healthy individuals in the GTEx database and tumor tissues in the TCGA database.

Significance analysis of expression differences was performed using un-paired Wilcoxon Rank Sum and Signed Rank Tests.

### 2.4 COX regression analysis and survival analysis

The study screened sample sources and obtained a high-quality prognostic dataset from the TCGA database from a TCGA-based predictive study previously published in the journal Cell (26). The TARGET follow-up data were obtained from the UCSC Cancer Genomics Browser (https://xenabrowser.net/datapages/) as a supplement. Samples with a follow-up period of<30 days were excluded from the analysis. A further log transformation was conducted for each expression value. Finally, the cancer types with<10 samples were also excluded. The expression data of 38 cancer types and the Disease-specific survival data of the corresponding samples were finally obtained.

The link between gene expression and prognosis in each cancer was analyzed by building a COX proportional hazards regression model utilizing the coxph function of R package survival (version 3.2-7). Moreover, the significance of the prognosis was obtained by statistical analysis using the Logrank test.

### 2.5 HTR2A, COMT, and PRODH regulate the tumor immune microenvironment by affecting immune infiltration in different cancers

The immune scores for each individual in each tumor were measured on the basis of gene expression utilizing the R package ESTIMATE (27). The immune scores for 10,180 tumor samples across 44 types of cancer were obtained through the use of the corr.test function of the R package psych (version 2.1.6). The Pearson’s correlation coefficient between genes and immune scores was calculated for each cancer to identify the significantly correlated immune infiltration scores. Furthermore, based on gene expression, the expression of 6 immune cells per individual in each cancer was re-evaluated using the Timer method of the R package IOBR (28, 29).

### 2.6 HTR2A, COMT, and PRODH are linked with TMB and MATH in some cancers

To explore the association between gene activity and mutations in specific cancer types, the relationships between TMB and MATH with HTR2A, COMT, and PRODH expression were examined. TMB and MATH are effective predictive biomarkers and indicators of immunotherapeutic response in various cancers.

TMB is strongly associated with the response to PD-1/PD-L1 inhibitors and can serve as a predictive marker for immunotherapy efficacy in certain tumor patients (30). In addition, we performed a correlation analysis between three target genes and three immune checkpoint genes related to PD-1/PD-L1, and the immune checkpoint genes were derived from the literature (31).

MATH is an effective way to depict the deviation in the distribution of minor allele frequency (MAF) values for loci that are specific to the tumor, indicating how much the MAF deviates from the overall MAF distribution for that particular sample.

A higher MATH value indicates more significant tumor heterogeneity. The Simple Nucleotide Variation dataset at Level 4 of all TCGA samples that were analyzed utilizing MuTect2 software (32) was retrieved from the Genomic Data Commons (GDC) database (https://portal.gdc.cancer.gov/). In addition, the TMB and MATH of each tumor were calculated utilizing the tmb function of the R package maftools (version 2.8.05). Finally, the TMB, MATH, and gene expression data of the samples were integrated, yielding expression data for 37 cancers.

### 2.7 Evaluation and validation of the prognosis prediction model

This research utilized R package glmnet to combine gene expression data, survival time and survival status. Regression analysis was performed utilizing the LASSO-COX technique. In addition, a 10-fold cross-validation was established to acquire the optimal model.

Following this, receiver operating characteristic (ROC) analysis was carried out using the R package pROC (version 1.17.0.1) to obtain the area under the curve (AUC). Specifically, the follow-up time and RiskScore scores of the patients were obtained, and the ROC analysis was carried out at the 365, 1095, 1825, and 3650 time points utilizing the roc function of pROC. The AUC and confidence interval (CI) were also assessed utilizing the ci function of pROC to determine the final AUC outcomes.

Subsequently, the optimal cutoff value of RiskScore was calculated utilizing the R package maxstat, and the logrank test method was utilized to assess the prognostic differences between different groups of samples.

Then, the predictive values of these features in the 598 samples were evaluated based on the COX method using the R package survival, R package rms, and the constructed nomogram.

Further, ROC and Kaplan-Meier (KM) analyses were performed using the CGGA dataset to re-validate the reliability of the prediction model.

### 2.8 Gene validation and immunocyte analysis in scRNA-seq data

The above studies could not confirm cell heterogeneity, so the scRNA-seq approach was used. The scRNA-seq dataset from the CGGA database was retrieved, and gene expression and immune cells in glioma were validated and evaluated utilizing the R packages dplyr, seurat, and patchwork.

### 2.9 Cluster analysis and cluster grouping for prognosis evaluation

Cluster analysis was performed using ConsensusClusterPlus, using agglomerative pam clustering with a 1-pearson correlation distances and resampling 80% of the samples for 10 repetitions. The optimal number of clusters was determined using the empirical cumulative distribution function plot.

### 2.10 qRT-PCR and IHC verification of candidate genes

As a verification group, we used T98G, U87 and U251 cell lines in order to determine the reliability of the research conclusions. For the qRT-PCR detection of HTR2A, COMT, and PRODH, we also used 293T as a control group, and GAPDH was used as the control gene. Magnetic beads and nucleic acid extraction were used to extract mRNA (BioPerfectus, Jiangsu, China). In order to amplify mRNA by sybr green fluorescent quantitative PCR, we used a one-step reverse transcription fluorescent quantitative kit (BBI lifesciences, Shanghai, China). The amplification instrument used was the BIOER Gene9660 fluorescent quantitative PCR instrument. In order to analyze the specificity of cDNA amplification, melt curves were used, and Amplification Data was used to analyze gene expression differences.

Moreover, we collected IHC results for HTR2A, COMT, and PRODH in human glioma tissue using The Human Protein Atlas (https://www.proteinatlas.org/).

## 3 Results

### 3.1 Differential expression of HTR2A, COMT, and PRODH in various tissues

**Figure 2:**
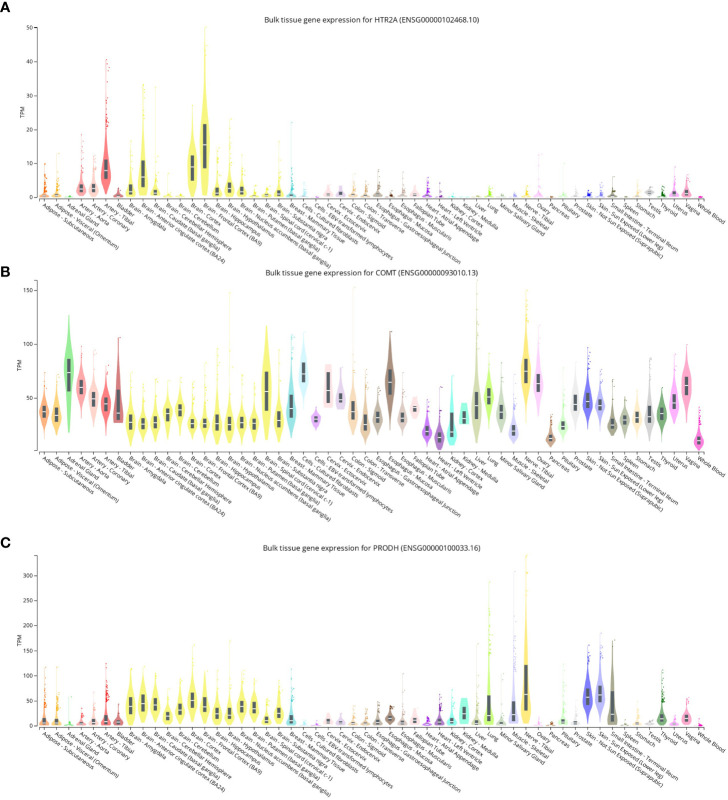
**(A-C)**: Expression levels of HTR2A, COMT, and PRODH in different tissues of healthy people.

It was found that the expression levels of HTR2A, COMT, and PRODH were determined to be similar in all tissues, but the brain clearly showed high expression, followed by the arteries (**Figures 2A-C**). HTR2A, COMT, and PRODH play a broad role between neuronal cell activity and neurotransmitters in the brain, and it is not unexpected that they have higher expression in the brain and arteries.

### 3.2 Differential expression of HTR2A, COMT, and PRODH in various cancers

**Figure 3:**
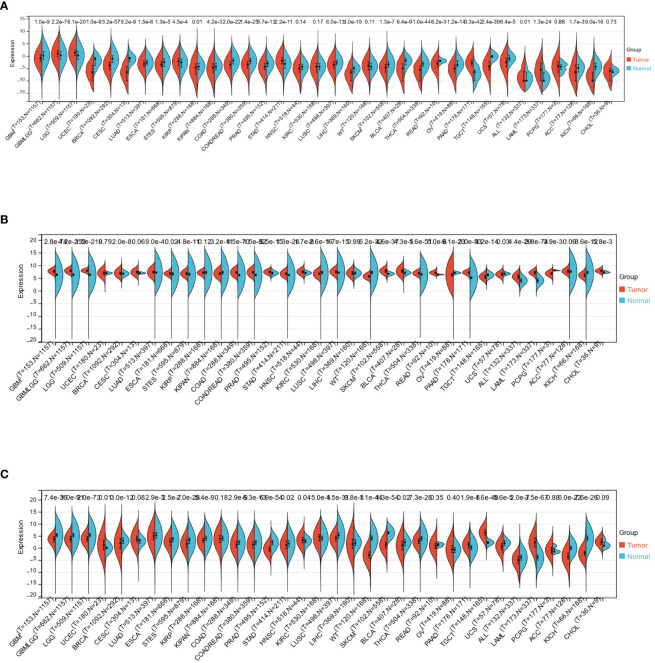
**(A-C)**: Expression differences of HTR2A, COMT, and PRODH in different cancer types.

This study observed significant upregulation of HTR2A in 7 cancers (GBMLGG、LGG、STES、STAD、PAAD、ALL、LAML) and significant downregulation in 22 cancers (GBM、UCEC、BRCA、CESC、LUAD、ESCA、KIRP、KIPAN、COAD、COADREAD、PRAD、LUSC、LIHC、SKCM、BLCA、THCA、READ、OV、TGCT、UCS、ACC、KICH)(**Figure 3A**). Also, considerable upregulation of COMT was observed in 21 cancers(GBM、GBMLGG、LGG、BRCA、LUAD、ESCA、STES、COAD、COADREAD、PRAD、STAD、HNSC、LUSC、SKCM、BLCA、THCA、READ、PAAD、ALL、LAML、CHOL) and significant downregulation in 8 cancers(KIPAN、KIRC、WT、OV、TGCT、UCS、PCPG、KICH) (**Figure 3B**). On the other hand, notable up-regulation of PRODH was observed in 5 cancers(UCEC、HNSC、KIRC、PAAD、TGCT). Its downregulation was witnessed in 22 cancers(GBM、GBMLGG、LGG、BRCA、LUAD、ESCA、STES、KIRP、COAD、COADREAD、PRAD、STAD、LUSC、LIHC、WT、SKCM、BLCA、THCA、UCS、ALL、ACC、KICH) (**Figure 3C**).

### 3.3 COX regression analysis and survival analysis

**Figure 4:**
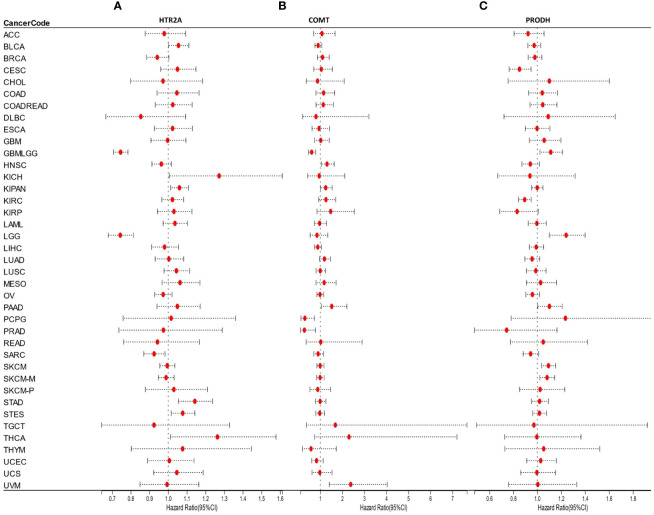
**(A-C)**: Prognostic performance of HTR2A, COMT, and PRODH in different cancer types.

It was found that the expression of HTR2A was high in 3 tumor types (supratentorial ependymomas [STES], pan-kidney [KIPAN], stomach adenocarcinoma [STAD]), predicting poor prognosis; however, it was poorly expressed in 2 tumor types (GBMLGG, low-grade glioma [LGG]) predicting the poor prognosis (**Figure 4A**).

COMT was highly expressed in 3 tumor types (KIPAN, uveal melanoma [UVM], pancreatic adenocarcinoma [PAAD]) with poor prognosis and poorly expressed in 2 tumor types (GBMLGG, pheochromocytomas, and paragangliomas [PCPG]) with poor prognosis (**Figure 4B**).

Moreover, the expression of PRODH was high in 4 tumor types (GBMLGG, TCGA-LGG, TCGA-skin cutaneous melanoma [SKCM], TCGA-SKCM-M) with poor prognosis and poorly expressed in 6 tumor types (cervical squamous cell carcinoma [CESC], lung adenocarcinoma [LUAD], kidney renal papillary cell carcinoma [KIRP], prostate adenocarcinoma [PRAD], kidney renal clear cell carcinoma [KIRC], lung squamous cell carcinoma [LUSC]) with poor prognosis (**Figure 4C**).

The optimal cutoff value for HTR2A was determined utilizing the R package maxstat. To obtain the best cutoff value, the minimum grouping sample size was set to >25% and the maximum grouping sample size was set to<75%.

According to this, individuals were sorted into two groups with high and low expressions respectively. The prognostic differences between the two groups were further analyzed utilizing the survfit function of the R package survival. The logrank test method was utilized to assess the significance of prognostic differences among samples from different groups.

**Figure 5:**
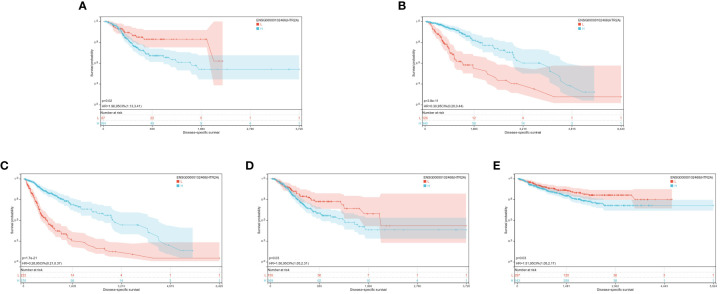
**(A-E)**: KM prognosis of HTR2A in TCGA-STAD, TCGA-LGG, TCGA-GBMLGG, TCGA-STES, TCGA-KIPAN.

Finally, significant prognostic differences were witnessed in GBMLGG (p = 1.7e-21), LGG (p = 3.8e-11), TCGA-STES (p = 0.03), KIPAN (p = 0.03), and STAD (p = 0.02) (**Figures 5A-E**).

**Figure 6:**
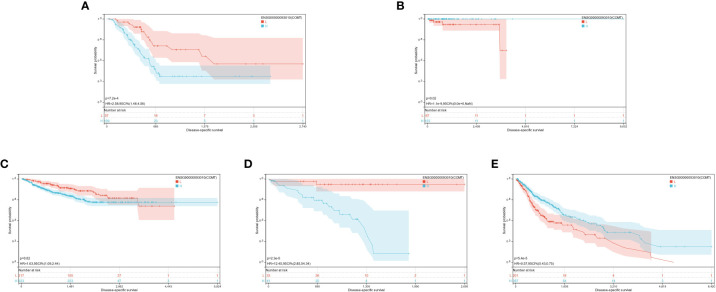
**(A-E)**: KM prognosis of COMT in TCGA-PAAD, TCGA-PCPG, TCGA-KIPAN, TCGA-UVM, TCGA-GBMLGG.

Based on the optimal cutoff values for COMT, the individuals were sorted into high and low-expression groups. The significant prognostic differences were demonstrated as follows: GBMLGG (p = 5.4e-5), KIPAN (p = 0.02), UVM (p = 2.3e-5), PAAD (p = 7.2e-4), and PCPG (p = 0.02) (**Figures 6A-E**).

**Figure 7:**
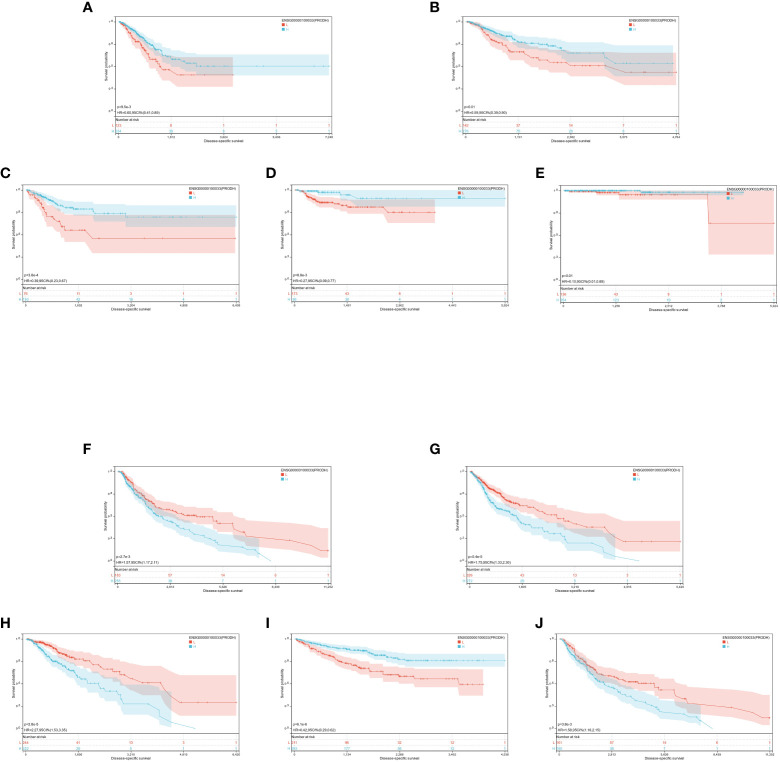
**(A-J)**: KM prognosis of PRODH in TCGA-LUAD, TCGA-LUSC, TCGA-CESC, TCGA-KIRP, TCGA-PRAD, TCGA-SKCM, TCGA-GBMLGG, TCGA-LGG, TCGA-KIRC.

The optimal cutoff value for PRODH was utilized to partition the individuals into high and low-expression groups. The results indicated significant prognostic differences in GBMLGG (p = 5.4e-5), LGG (p = 2.8e-5), CESC (p = 3.8e-4), LUAD (p = 9.5e-3), KIRP (p = 8.8e-3), PRAD (p = 2.3e-4), KIRC (p = 6.1e-6), LUSC (p = 0.01), SKCM (p = 2.7e-3), and SKCM-M (p = 3.8e-3) (**Figures 7A-J**).

It was found that the expression of three genes (HTR2A, COMT, and PRODH) had significantly different effects on the prognosis of multiple cancers. Different genes play different roles for different cancers, and the same gene may have different prognostic effects for various cancers. However, it was demonstrated that they all had significant prognostic differences in GBMLGG. Additionally, the low expression of HTR2A and COMT indicated unfavorable prognosis, while the high expression of PRODH corresponded to a worse prognosis. This was just opposite to the trend of their differential expression in each cancer compared with the normal group, which was further explored in the following studies.

### 3.4 HTR2A, COMT, and PRODH regulate TIME by affecting immune infiltration in different cancers

**Figure 8:**
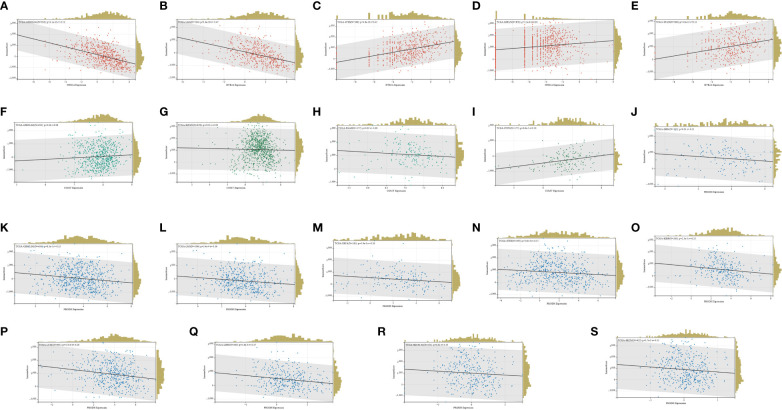
**(A-E)**: HTR2A immune infiltration in TCGA-GBMLGG, TCGA-LGG, TCGA-STES, TCGA-KIPAN, TCGA-STAD; **(F-I)**: COMT in TCGA-GBMLGG, TCGA-KIPAN, TCGA-PAAD, Immune infiltration in TCGA-PCPG; **(J-S)**: PRODH in TCGA-GBM, TCGA-GBMLGG, TCGA-LGG, TCGA-ESCA, TCGA-STES, TCGA-KIRP, TCGA-LUSC, TCGA-LIHC, TCGA-SKCM, Immune infiltration in TCGA-OV.

HTR2A expression was strongly linked with immune infiltration in 25 cancers, of which 21 were significantly positively associated, and 4 were significantly negatively associated. Five cancers that ranked high in the immune scores for significant differences in the COX regression and survival analysis were selected (**Figures 8A-E**).

In terms of the COMT expression, it was significantly associated with immune infiltration in 19 cancers, 13 of which were positively associated and 6 of which were negatively associated. Four of the cancers with significant differences in the COX regression and survival analysis ranked high in the immune scores (**Figures 8F-I**).

Moreover, significant association was identified between PRODH expression and immune infiltration in 18 cancers, of which 8 showed positive association and 10 showed negative association. Ten of the cancers with significant differences in COX regression and survival analysis were ranked high in the immune scores (**Figures 8J-S**).

**Figure 9:**
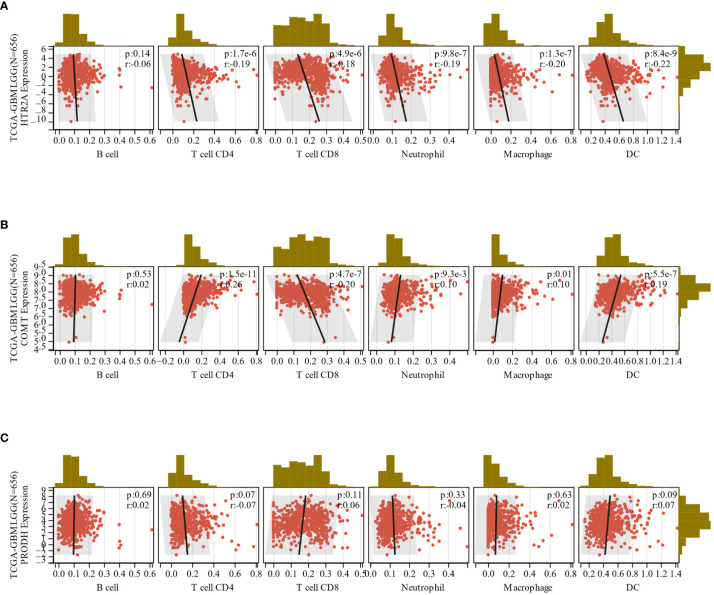
**(A-C)**: The correlation between the expression of HTR2A, COMT and PRODH in GBMLGG and the infiltration of 6 immune cells.

In total, 9406 tumor samples from 38 tumor types were scored for 6 types of immune cell infiltration. The Pearson’s correlation coefficient between genes and immune cell infiltration scores in each tumor was determined utilizing the corr.test function of R package psych (version 2.1.6) to identify significantly correlated immune cell infiltration scores. In 39 cancer types, including GBMLGG, HTR2A, COMT, and PRODH expressions were significantly correlated with immune cell infiltration (**Figures 9A-C**; **Supplementary Table 1**).

### 3.5 HTR2A, COMT, PRODH are linked with TMB and MATH in some cancers

**Figure 10:**
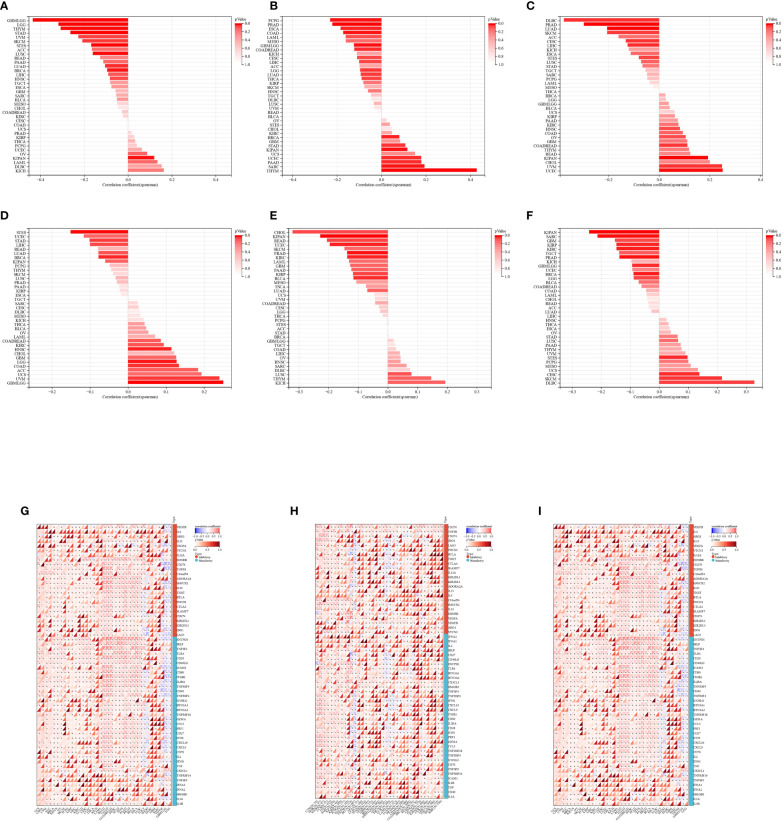
**(A-C)**: Spearman correlation of HTR2A, COMT, PRODH to TMB of various cancer types; **(D-F)**: Spearman correlation of HTR2A, COMT, PRODH to MATH of various cancer types; **(G-I)**: HTR2A, COMT, PRODH to each cancer Spearman correlation of genes related to PD-1/PD-L1 immune checkpoint in various cancer types "*": P<0.05.

The correlation between TMB and HTR2A, COMT, and PRODH was examined for each cancer using Spearman’s correlation method. The findings demonstrated that HTR2A was significantly correlated in 11 tumors, with a significant positive correlation in KIPAN and a significant negative correlation in 10 tumors (GBMLGG, LGG, LUAD, BRCA, STES, STAD, LUSC, THYM, SKCM, and UVM) (**Figure 10A**).

The significant correlation of COMT was noted in 15 tumors, with positive correlation in 7 tumors (BRCA, SARC, KIPAN, STAD, UCEC, THYM, and PAAD) and negative correlation in 8 tumors (GBMLGG, LGG, LUAD, COAD, COADREAD, ESCA, PRAD, and PCPG) (**Figure 10B**).

Also, PRODH was considerably associated in 10 tumors, with significant positive correlation in 4 tumors (COADREAD, KIPAN, UCEC, and UVM), and significant negative correlation in 6 tumors (CESC, LUAD, PRAD, LIHC, SKCM, and DLBC) (**Figure 10C**).

With regard to the Spearman’s correlation with MATH, HTR2A was significantly correlated in 7 tumors, with significant positive correlation in 5 tumors (GBMLGG, LGG, COAD, HNSC, and UVM), and significant negative correlation in 2 tumors (BRCA, STES) (**Figure 10D**).

Significant negative correlation of COMT with MATH was observed in 6 tumors (KIRP, KIPAN, PRAD, UCEC, KIRC, and BLCA) (**Figure 10E**).

PRODH was significantly correlated in 11 tumors, with 4 tumors (CESC, STES, SKCM, and DLBC) showing positive correlation and 7 tumors (GBMLGG, BRCA, SARC, KIRP, KIPAN, PRAD, and KIRC) showing negative correlation (**Figure 10F**).

Importantly, significant negative correlation of HTR2A and COMT was found with the TMB genomic heterogeneity in GBMLGG, while the correlation of PRODH was not significant.

HTR2A showed a significant positive correlation in the MATH genomic heterogeneity of GBMLGG, PRODH showed the opposite (significant negative correlation), while COMT was not significantly correlated.

Additionally, we conducted a correlation analysis on the genes related to HTR2A, COMT, PRODH, and PD-1/PD-L1 immune checkpoints and found that HTR2A is related to 51 genes in GBMLLGG, of which 8 are positively correlated and 43 are negatively correlated. In GBMLLGG, COMT was correlated with 23 genes, of which 16 were positively correlated and 7 negatively correlated. Among the 25 genes correlated with PRODH in GBMLLGG, 16 were positively correlated and 9 were negatively correlated. (**Figure 10G-I**; **Supplementary Table 2**)

### 3.6 Evaluation and validation of the prognosis prediction model

Three genes were obtained by setting the Lambda value to 0.00398195949408132. The constructed model equation was:


RiskScore=−0.374678180494389*HTR2A0.500208354524312*COMT+0.297301353392057*PRODH.

**Figure 11:**
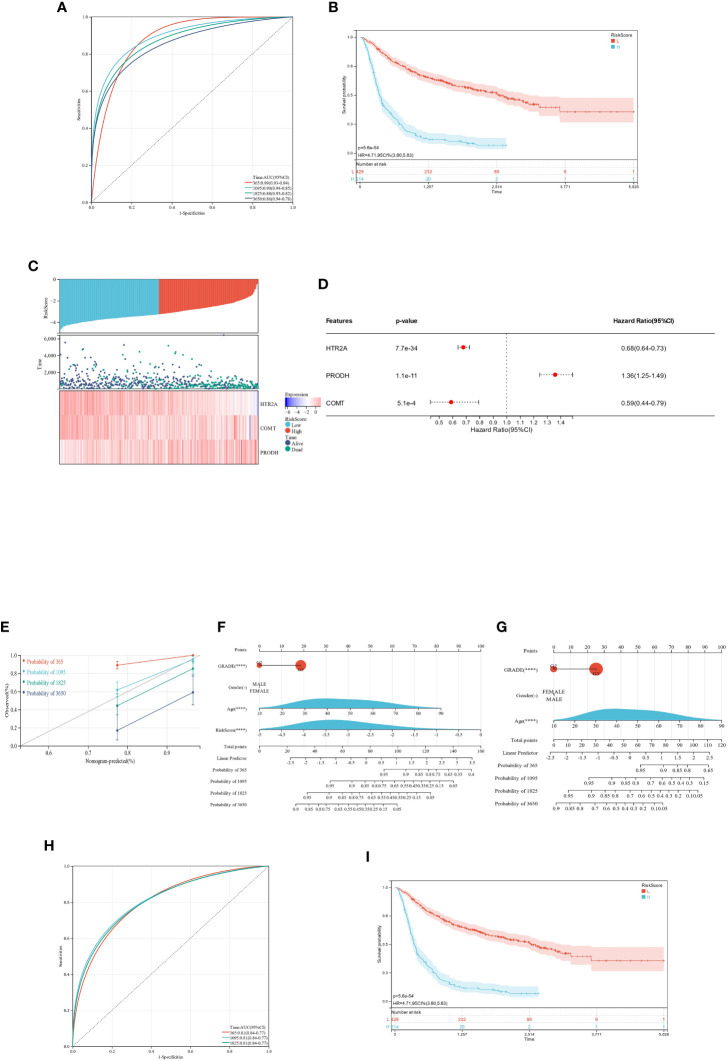
ROC and KM survival analysis for the prognostic difference of HTR2A, COMT, and PRODH in TCGA-GBMLGG patients. **(A)**: ROC curve of RiskScore model for survival and prognosis of TCGA-GBMLGG patients; **(B)**: KM curve of RiskScore model for survival and prognosis of TCGA-GBMLGG patients; **(C)**: Patient survival significantly decreases with increasing RiskScore; HTR2A and COMT expression is downregulated with increasing RiskScore, while PRODH expression is upregulated; **(D)**: COX regression analysis of the prognostic significance of the expression of 3 genes in 598 samples; **(E)**: Nomogram calibration curve of the prediction model (containing 4 features [grade, gender, age, RiskScore]) for the survival and prognosis of GBMLGG patients; **(F)**: Nomogram column line plot of prediction model (containing 4 features [grade, gender, age, RiskScore]) on the survival and prognosis of GBMLGG patients; **(G)**: Nomogram column line plot of prediction model (containing 3 features [grade, gender, age]) on the survival and prognosis of GBMLGG patients; **(H)**: ROC curves of the RiskScore model on the survival and prognosis of CGGA-GBMLGG patients; **(I)**: KM curves of the RiskScore model on the survival and prognosis of CGGA-GBMLGG patients. "-": P ≥ 0.05 ;"****": P<0.001.

The ROC curves constructed from this model were utilized to evaluate the predicted 1-, 3-, 5-, and 10-year survival for the TCGA-GBMLGG patients, yielding AUC-1 year = 0.89, AUC-3 years = 0.90, AUC-5 years = 0.88, and AUC-10 years = 0.86 (**Figure 11A**).

By ensuring that the minimum grouping sample size was >25% and the maximum grouping sample size was<75%, the optimal cutoff value for RiskScore was calculated.

The obtained optimal cutoff value was 0.324557969693656, according to which the individuals were sorted into high and low-expression groups. Subsequently, the significance of the prognostic difference between different groups of samples was further explored, and the results validated a significant prognostic difference (p = 8.6e-20) (**Figure 11B**).

The link between various risk scores and the duration of follow-up, occurrences of events, and changes in gene expression for each individual was analyzed in this study. The study found a significant decrease in the survival rate of individuals as the risk scores increased. As expected, the HTR2A and COMT genes were protective factors and showed a reduction in expression with increasing risk scores. The PRODH gene was a risk factor showing upregulation in expression with increasing risk score (**Figure 11C**). The performance was consistent with the findings described above.

The prognostic significance of survival time, survival status, and the expression of 3 genes were assessed in 598 samples through the integration of data using the COX method.

The overall prognostic difference was significant (logtest = 3.77661553973387e-30, sctest = 4.26338627161977e-36, waldtest = 5.60752151601653e-34), with a C-index of 0.752420921507274. All three genes highlighted significant prognostic differences in the samples, and the trend of correlation was consistent with the expected outcome (**Figure 11D**).

Using the integrated data on survival time, survival status, and four features (GRADE, Gender, Age, RiskScore), a nomogram was developed utilizing the COX method. It was used to evaluate the prognostic significance of these features in a sample of 598. The overall C-index of the model was 0.832101597009854, the 95% CI was (0.79949380759634-0.864709386423367), and the p value = 1.18589136333727e-88 (**Figures 11E, F**).

Another nomogram was developed utilizing the COX method based on the integrated data on survival time, survival status, and 3 features (grade, gender, and age) to evaluate the prognostic significance of these features in 598 samples. The overall C-index, 95% CI, and p value of the model was 0.788265414977598, (0.743015146095509-0.833515683859686), and 8.91421287369874e-36, respectively (**Figure 11G**).

The ROC curves constructed using this model were validated for 1-, 3-, and 5-year survival analysis for CGGA-GBMLGG patients, yielding AUC-1 year = 0.81, AUC-3 years = 0.81, and AUC-5 years = 0.81 (**Figure 11H**).

The individuals were partitioned into high and low-expression groups on the basis of the best cutoff value of RiskScore in CGGA-GBMLGG. The analysis results verified the significant prognostic differences between the samples of distinct groups (p = 5.6e-54). (**Figure 11I**)

It was found that the prediction of 1-, 3-, 5-, and 10-year survival of GBMLGG patients was better in the model with RiskScore added (C-index: 0.832101597009854) than in the model without RiskScore added (C-index: 0.788265414977598). Moreover, the ROC and KM analyses of this model demonstrated good results in two independent datasets.

Therefore, it was speculated that HTR2A, COMT, and PRODH, which play key roles in SCZ, also have critical effects on the progression of multiple tumors and the prognosis of patients, especially in GBMLGG. SCZ and its related gene expression were highly correlated with multiple tumors, especially GBMLGG. The model constructed using the three genes showed promising results in predicting the survival of GBMLGG patients.

### 3.7 scRNA-seq data validation and immunocyte analysis

**Figure 12:**
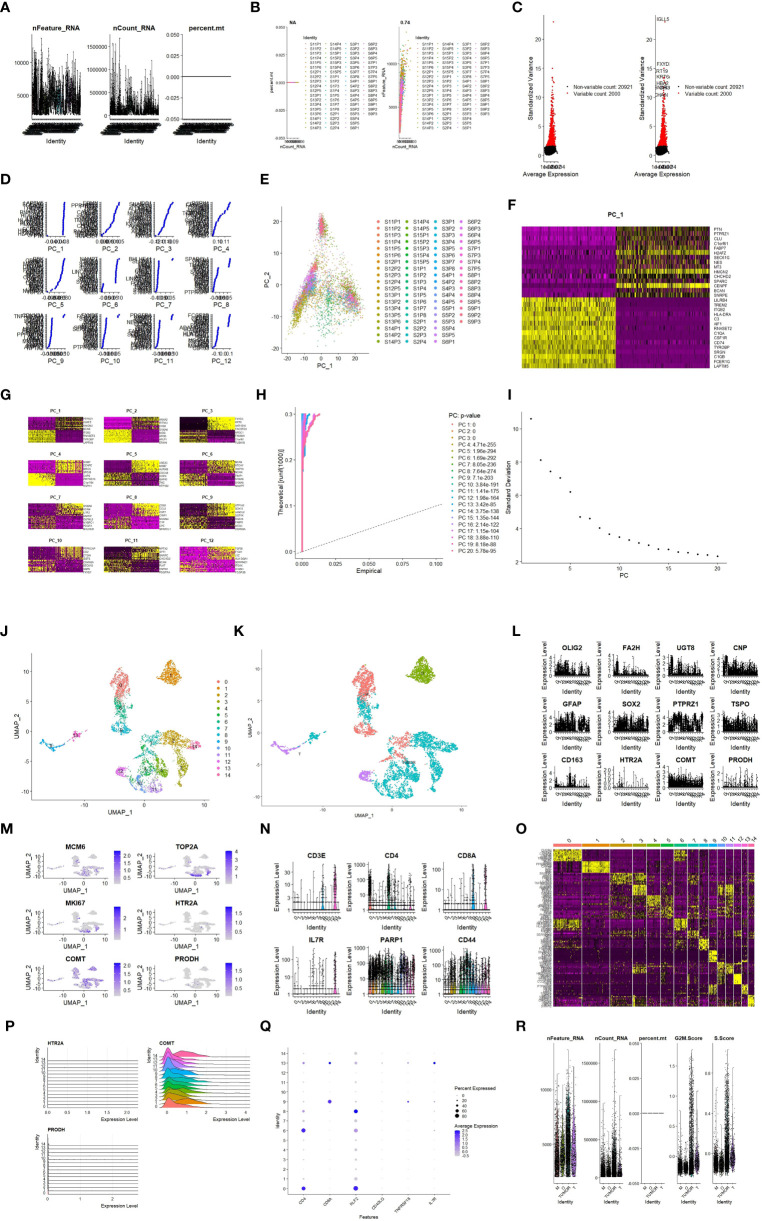
scRNA-seq data validation and immunocyte analysis. **(A)**: Quality control visualization (nFeature_RNA expression number, nCount_RNA cell number); **(B)**: Quality control visualization (relationship between nFeature_RNA expression number, nCount_RNA cell number, per cent.mt mitochondrial ratio); **(C)**: The Top 10 high heterogeneity genes in top2000 DEGs; **(D)**: The specific genes in 12 PCs; **(E)**: PCA results; **(F)**: Genes included in the PCA for PC1; **(G)**: Genes included in each PC of PCA; **(H)**: PC curves and p values showing data dimensions; **(I)**: Data dimensions determined based on inflection points; **(J)**: U-map subcluster plot; **(K)**: Cluster annotation; **(L)**: Expression of certain genes in each group; **(M)**: Expression of certain genes in each group with the minimum and maximum display value; **(N)**: Expression of immune markers in each group; **(O)**: Specific genes in each group; **(P)**; Expression of specific genes in each group; **(Q)**; Expression of specific genes in each group; **(R)**: Cell cycle of each group.

The studied genes and immune infiltrating cells were further analyzed in scRNA-seq data, starting with the normalization of scRNA-seq information and quality control visualization (**Figures 12A, B**). Thus, the differentially expressed genes (DEGs) and top high heterogeneity genes in each principal component (PC) were identified (**Figures 12C-I**). Subsequently, annotations were added to Cluster, and the data on the expression of target DEGs and immune-related genes (IRGs) in each group were processed and visualized (**Figures 12J-R**).

The study revealed that COMT was expressed at high levels in all cell lines of GBMLGG patients, HTR2A showed more overexpression in tumor cells, and PRODH exhibited more overexpression in T cells (**Figures 12I-M**). It may explain the finding that HTR2A had a positive correlation in GBMLGG patients compared to normal controls but a negative correlation with patient survival and prognosis. PRODH showed an opposite effect, as the degree of enrichment of T cells, especially CD8^+^ T cells, in the GBMLGG patients affected patient prognosis. Consequently, scRNA-seq analysis of IRGs was conducted, with a specific focus on CD8^+^ T cells that were more notable in the above study. The results found that CD8A was highly expressed in the T cell group (**Figures 12N-Q**).

### 3.8 Cluster analysis and prognostic assessment of clustering groups

**Figure 13:**
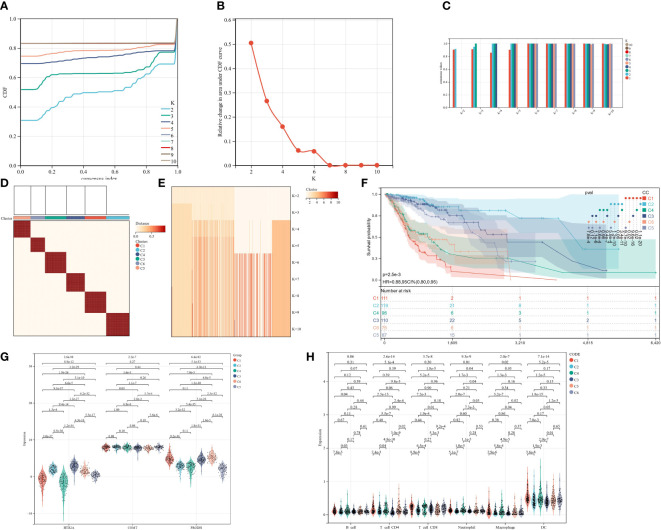
Cluster analysis and prognostic assessment of clustering groups. **(A)**: CC cumulative distribution curve; **(B)**: Area under CC distribution curve; **(C)**: CC sample clustering consensus; **(D)**: CC clustering heatmap; **(E)**: CC sample clustering consensus; **(F)**: KM prognosis analysis; **(G)**; Expression differences of 3 genes in different CC groups; **(H)**; Expression differences in 6 subtypes of immune cells in different CC groups.

Finally, the cluster analysis was performed for the data of TCGA-GBMLGG. The K = 6 (**Figures 13A-E**) was selected based on the area under the cumulative distribution function (CDF) curve line being as large as possible, the CDF Delta decrease being as slow as possible, and the intra-group consistency.

The prognostic differences between the samples of different groups were evaluated utilizing the survfit function of the R package survival. The significance of the prognostic differences between the samples of different groups was assessed utilizing the logrank test method. Ultimately, significant prognostic differences were observed in almost all pairwise comparisons in the 6 groups clustered by CC. (**Figure 13F**). Separate analyses were also conducted for the expression differences of the three target genes and 6 subtypes of immune cells in different CC clusters. The results showed that almost all had statistically significant differences (**Figures 13G, H**).

### 3.9 qRT-PCR and IHC verification of candidate genes

As a means of verifying the reliability of the conclusions, we performed qRT-PCR.

**Figure 14:**
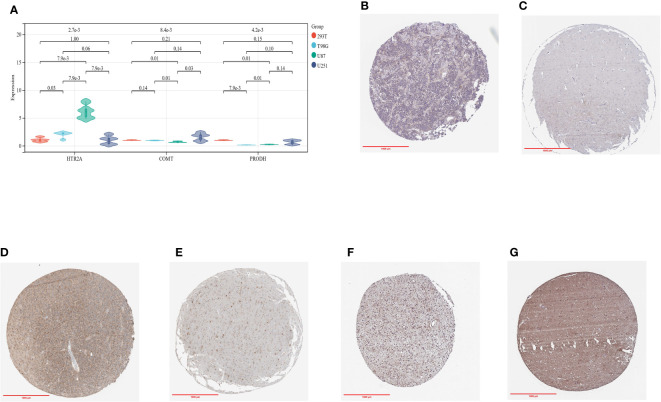
**(A)**: qRT-PCR results in three glioma cell lines and control cell lines; **(B)**: IHC of HTR2A in GBMLGG tumor samples; **(C)**: IHC of HTR2A in control samples; **(D)**: IHC of COMT in GBMLGG tumor samples; **(E)**: IHC of COMT in control samples; **(F)**: IHC of PRODH in GBMLGG tumor samples; **(G)**: IHC of PRODH in control samples.

The expression of HTR2A and PRODH differed significantly between T98G and U87, with HTR2A being more highly expressed than the control group, while PRODH was less expressed (**Figure 14A**). Based on our analysis of the ChIP dataset, this is consistent with what we found above. A significant difference was not observed among the three genes in U251, which may be the result of U251’s poor chemotaxis. Further studies will verify this hypothesis.

In addition, we evaluated the IHC results of the three genes HTR2A, COMT and PRODH in the online data set from human glioma tissue samples, and their differences were evident (**Figure 14B-G**).

## 4 Discussion

The present study found that HTR2A, COMT, and PRODH played a broad role between neuronal cell activity and neurotransmitters in the brain, and thus their levels of expression were high in the brain than in other tissues. Significant upregulation of HTR2A was observed in 7 tumors and significant downregulation in 22 tumors. Moreover, HTR2A had high expression in three tumor types (STES, KIPAN, and STAD) with poor prognosis and had low expression in two tumor types (GBMLGG, LGG) with poor prognosis. The upregulation of COMT was observed in 21 tumors and significant downregulation in 8 tumors. Elevated expression of COMT in KIPAN, UVM, and PAAD was linked to an unfavorable prognosis, while reduced expression of COMT in GBMLGG and PCPG was linked to a poor prognosis.

Furthermore, the significant upregulation of PRODH was observed in 5 tumors and significant downregulation in 22 tumors. PRODH had elevated expression in 4 tumor types (GBMLGG, LGG, SKCM, and SKCM-M) with poor prognosis and low expression in 6 tumors (CESC, LUAD, KIRP, PRAD, KIRC, and LUSC).

Herein, a preliminary conclusion could be reached that the effect of SCZ-related genes HTR2A, COMT, and PRODH was highly variable in different cancers, and the prognostic significance of SCZ on cancer development needed to be explicitly explored for various cancers.

According to the current research findings, a strong association was found between the expression of SCZ-related genes HTR2A, COMT, and PRODH and the prognosis of GBMLGG patients. As reported previously, HTR2A has a higher degree and is closely associated with neuroactive ligand-receptor interactions, and it is downregulated in U251 cells (33). Previous studies have shown that HTR2A expression is associated with low-grade and high-grade gliomas through neuroactive ligand receptors (34). In this study, we used three cell lines for verification. We found that HTR2A was up-regulated in T98G and U87, which was completely consistent with our research expectations, but we did not find any differences in U251. The reason we speculate may be related to U251 cells’ weak invasiveness, which needs further study. COMT, found in mature and developing oligodendrocytes, is responsible for metabolizing dopamine. A common polymorphism in COMT, the Val (G) variant at position 158 (rs4680, COMT G/A), results in lower thermal stability and, thus, more dopamine degradation (35). The major functional domain changes in COMT are memory (such as delayed memory, list recall, graphical recall, and word recall) (36). Therefore, low COMT expression leads to a reduced ability of patients to return to work, causing an unfavorable prognosis. In addition, PRODH has also been associated with cognition, and it is a putative tumor suppressor. Limited evidence exists regarding the correlation between this gene and GBMLGG. However, a study by Eduard et al. found that lower, higher levels of PRODH in GBM predicted poorer survival (37), which is consistent with the current research findings.

In the investigation of immune scores and immune cell infiltration, correlation analysis was performed with three genes related to the occurrence and prognosis of GBMLGG, resulting in several significant and noteworthy findings.

As demonstrated in the results of COX regression and survival analysis, HTR2A and COMT had the same trend of expression in GBMLGG (high expression in the positive group). In contrast, PRODH had the opposite result (low expression in the positive group). In the analysis of their roles in survival prognosis, it was found that high expression of HTR2A and COMT led to a good prognosis, while the opposite was true for PRODH, whose high expression may lead to a poor prognosis.

It was then found that there was a consistent correlation result for HTR2A and PRODH (negative correlation) and the opposite correlation for COMT (positive correlation) in the immune cell infiltration analysis of GBMLGG. Moreover, it was found that all three genes indicated significant correlations in the immune cells (p< 0.05), although the direction of correlation varied among the 6 subtypes of immune cells. The specific outcomes suggested that HTR2A was negatively correlated in all 6 subtypes of immune cells, which was consistent with the results of the effect of this gene on the survival and prognosis of GBMLGG. Additionally, COMT showed a negative correlation as HTR2A only in CD8^+^ T cells and a positive correlation in the remaining five subtypes of immune cells, which was consistent with the positive correlation of this gene with the immune scores of GBMLGG. In contrast, PRODH showed a positive correlation (including CD8^+^ T cells) except for a negative correlation in CD4^+^ T cells, which also concurred with the effect of this gene on the survival and prognosis of GBMLGG.

In this study on the correlation of genomic heterogeneity of three genes with GBMLGG, it was found that HTR2A and COMT presented a significant negative correlation in TMB genomic heterogeneity. TMB is strongly linked with the efficacy of PD-1/PD-L1 inhibitors, which may provide a prediction for the immunotherapy efficacy by TMB markers to some extent. The expression of these two genes might inversely affect the efficacy of immunotherapy through the TMB pathway and further influence patient survival and prognosis. Such results are in line with findings of this study. The emerging TMB biomarker shows promise as a potential indicator of immunotherapy response. It may be useful for predicting which patients could benefit from immune checkpoint inhibitor therapy (ICI) (38). An elevated TMB can increase the number of neoantigens that can attract the adaptive immune system, potentially serving as a useful prognostic indicator for the effectiveness of immunotherapy.

Over the past few years, multiple human cancers have shown a correlation between elevated TMB and clinical benefits. However, the TMB cutoffs linked to increased survival vary significantly between different types of cancer, indicating that there might not be a standardized definition of what constitutes high TMB (39).

HTR2A showed a significant positive correlation in the genomic heterogeneity of MATH in GBMLGG, while PRODH showed the opposite (significant negative correlation). MATH effectively represents the deviation of the distribution of MAF values at tumor-specific loci, which corresponds to the extent to which MAF deviates from the overall distribution of MAF in that sample. Higher MATH values reflect elevated tumor heterogeneity. The HTR2A expression may positively reflect the degree of tumor heterogeneity through MATH and further influence patient survival and prognosis. The negative correlation of PRODH showed the opposite effect, and this result was obviously in line with the findings of this study. In a study of glioma patients, Wu et al. (40) found that gene mutation patterns and signaling pathway enrichment analysis results were much more complex in the high MATH group than in the low MATH group. The recurrence intervals tended to be shorter in the high MATH group then the low MATH group. Furthermore, mutations in individuals with high MATH values were enriched in the “BH3 anti-apoptotic”, “MAD2 inhibitory signaling”, and “glutathione biosynthesis” signaling pathways, which conferred anti-apoptotic effects (41).

As a result of the above studies, we have demonstrated that schizophrenia-related genes (HTR2A, COMT, PRODH) are significantly related to the development and occurrence of various cancers, but the directionality of each cancer differs. In GBMLGG, all three genes have good predictive effects, and their mechanisms may be related to immune pathways (especially T cell CD8) and genomic heterogeneity (especially TMB and MATH). Last but not least, the model we constructed using these three genes was highly predictive of GBMLGG survival. Our prediction model was then verified in CGGA-GBMLGG and we achieved better results.

In addition, in the glioma single-cell sequencing data set, we verified and analyzed the above studies on gene expression and immune cells, and the results were consistent with our conclusions.

Finally, we performed unsupervised CC clustering in TCGA-GBMLGG using three genes, and the grouped prognostic analysis and differential analysis showed that the CC clustering of the three genes can well group the risk of glioma patients after rain, and that such effects are associated with an infiltration of immune cells.

According to the results of our investigation, the prognostic level is closely correlated with the variations in the expression of the schizophrenia-related genes HTR2A, COMT, and PRODH in various malignancies. This shows that these genes might be useful markers for determining a patient’s prognosis and providing them with tailored care. Additional research could examine these genes as elements of prognostic prediction models and evaluate their applicability in clinical settings.

Overall, this study offers fresh information and insights for the investigation of genes linked to schizophrenia in the field of cancer. In addition to providing a theoretical foundation for the development of individualized therapy and immunotherapy, further study will also help to shed light on the precise mechanism by which these genes function in the occurrence, development, and treatment of cancer. It will also offer new guidance for the prognosis assessment and treatment selection of cancer patients.

## 5 Conclusion

In various cancer types, schizophrenia-related genes (HTR2A, COMT, and PRODH) have different expression patterns, and the direction of down-regulation varies. In the analysis of GBMLGG, we observed that the upregulation of HTR2A and COMT was significantly positively correlated with the occurrence of the disease, contrary to PRODH; however, the upregulation of HTR2A and COMT was significantly negatively correlated with the survival of the disease, contrary to PRODH. Based on the analysis of immune cells, we observed that abnormal expression of three genes was associated with the infiltration of T cells CD8, which may play an important role in the survival of GBMLGG. Through the TMB pathway, the expression of HTR2A and COMT may reverse the effect of immunotherapy and affect a patient’s prognosis and survival. The expression of HTR2A may contribute to the degree of tumor heterogeneity through MATH, as well as affecting the patient’s prognosis and survival. In contrast, PRODH has a negative correlation. As a final step, we evaluated and verified the predictive value of the survival prediction model we constructed.

## Data availability statement

The original contributions presented in the study are included in the article/**Supplementary Material**. Further inquiries can be directed to the corresponding author/s.

## Author contributions

YF and JS wrote the main manuscript text. PW is responsible for the main experiment. QW, FL and HX were responsible for proofreading and conception, and all authors reviewed the manuscript.
